# Doxastic Precautionary Principle as Political Encroachment

**DOI:** 10.1007/s12136-025-00642-6

**Published:** 2025-05-23

**Authors:** Maciej Juzaszek

**Affiliations:** 1 Centre for Legal Education and Legal Theory, University of Wroclaw, Uniwersytecka St, 22/26, 50-145 Wroclaw, Poland; 2 Faculty of Law and Administration, University of Silesia, Bankowa St, 11B, 40-007 Katowice, Poland

**Keywords:** Ethics of belief, Precautionary principle, Moral encroachment, Pragmatic encroachment, Evidentialism, Pragmatism, Political encroachment, Political minimalism, Purism

## Abstract

This article aims to reconcile the intuitions grounding two important positions from the ethics of belief: epistemic purism and reason pragmatism. They can conflict, especially at the level of what we ought to believe all-things-considered. They manifest themselves in two important meta-principles that constrain law and policy-making that seem to be in tension as well. The first is the principle of evidence-based regulation, which says that legal rules should only be based on current scientific knowledge. The second is the precautionary principle, according to which authorities should regulate (or even prohibit) an activity that may cause harm to humans or the environment, even if there is insufficient scientific evidence to support such a claim. However, I argue that the precautionary principle can be interpreted from the perspective of ethics of belief as political encroachment on evidence-based regulation. As such, it can reconcile both the epistemic and pragmatic intuitions underlying these two principles.

## Problem Outline

The problem that I would like to address in this paper is the tension between the epistemic interpretations of two normative ideals: the *precautionary principle* (PP) and the *evidence-based approach to legal regulation* (EBR). Usually, the PP and the EBR are considered principles for regulatory action. However, I would like to focus on a step earlier — the epistemological level, from the perspective of the ethics of belief. According to the EBR, the law should reflect the state of contemporary science. To make a legislative decision about *X*, the legislative institution must have a set of assumptions, beliefs or other kinds of propositional attitudes about *X*^1^. Therefore, its beliefs should reflect the state of contemporary science, according to what I call *doxastic evidence-based approach to legal regulation* (dEBR). So, if science claims that *X*, then the legislative institution should believe *X*. But if science claims that it has no evidence for the existence of *X*, then the legislative institution should not believe *X* (usually, it means it should withhold belief in *X*). For example, if there is no scientific evidence for the existence of body snatchers, then (based on the dEBR) the legislative institution should not believe that there are body snatchers. And consequently (based on the EBR), it should not create regulations based on the belief that body snatchers exist.

In contrast to the EBR, the PP allows for the regulation of certain areas even if there is no scientific evidence for such regulation or evidence is insufficient. The main purpose of the PP is the protection of important social values, such as public health or the natural environment, from potential hazards that do not have a scientifically proven risk. If there had been confirmation, the legislative institution would have been able to regulate such a dangerous activity based on the dEBR and the EBR. Before applying the PP, the legislative institution should be convinced that the regulated phenomenon can present danger, but there is no scientific evidence about the details of the risk. Therefore, I assume that, by analogy with the dEBR, we can refer to the *doxastic precautionary principle* (dPP), operating at the level of the lawmaking institution’s beliefs.

If, according to the dEBR, lawmaking institutions should ground their beliefs about *X* solely in scientific evidence, and the dPP allows the belief that *X* is dangerous even if there is insufficient evidence to support such a belief, then the dEBR and the dPP may, at least prima facie, be in conflict. This conclusion seems paradoxical given that both are manifest in many legal systems, both national and international. I argue that this conflict is a derivative of the intuitions underlying epistemic purism and reasoned pragmatism — the positions from the ethics of belief that I believe underlie the dEBR and the dPP. The main question is whether it is possible to provide such an interpretation of the dEBR and the dPP that reconciles the conflicting intuitions. The normative answer from the field of ethics of belief may be to treat the dPP as a political encroachment on the dEBR.

## Ethics of Belief

Let me begin by introducing the basic framework of ethics of belief underlying my paper. Ethics of belief investigates various types of norms that govern the acceptance and maintenance of beliefs. It raises questions such as: ‘What should we believe?’ and, more generally, ‘On what basis should we adopt beliefs?‘ Therefore, the purpose of ethics of belief is not to describe what beliefs people hold or explain the underlying mechanisms responsible for why people believe something (as it is a task for psychology, experimental philosophy or sociology). It is a normative discipline that guides individuals in making morally and epistemically sound decisions regarding their beliefs rather, and it provides criteria for evaluating such decisions from a moral and epistemological point of view.

Contemporary literature recognizes that not only individuals, but also collective agents and institutions can possess knowledge, hold beliefs or even lie (see, e.g. Lackey, 2020). For example, people may ascribe beliefs to groups such as ‘European Council believes that pronouncing this plan may harm underage consumers’. If this is permitted, then collective agents should be subject to moral and epistemic evaluations according to, what I call, institutional ethics of belief. In this paper, I will explore the *ethics of belief for lawmaking institutions*. Particularly, I will focus on the question of how lawmaking institutions should adopt beliefs in situations that may potentially lead to great danger or harm, but where the risks (both the consequences and their probabilities) are uncertain. Should they wait for more detailed data provided by science to allow for the calculation of risks and cost-benefit analysis, or should they believe that such a danger requires precautionary steps?

One could raise a fundamental question: Why should we even be concerned with the ethical evaluation of lawmaking institutions’ beliefs rather than solely focusing on their actions (legislative process or enacted legal regulations)? There are at least two answers to this question. First, beliefs serve as reasons for action. If the lawmaking institution justifies legal regulation R by grounding it in belief B, but B is unjustified from an ethical standpoint, we can legitimately question the ethical justification of R itself. Second, some researchers argue that simply holding certain beliefs can be morally wrong, even if they do not directly harm anyone (see Basu, 2019). For instance, racist beliefs are considered wrong regardless of whether they lead to racist behaviour. Therefore, ethical reflection should encompass not only actions but also beliefs.

In the literature on the ethics of belief, two main approaches are traditionally distinguished: *evidentialism* and *pragmatism*. The former refers to William Clifford’s (1886, p. 346) famous principle that *it is wrong always, everywhere and for anyone to believe anything on insufficient evidence*, while the latter is inspired by William James’ (1979) *will to believe*. Contemporary ethics of belief take a more analytical approach, distinguishing between *epistemic purism* and *reasons pragmatism*. The former says that only alethic considerations can be relevant to what one (epistemically) ought to believe. The latter, on the other hand, claims that practical (and ethical) considerations constitute non-alethic reasons that are relevant to what one (pragmatically) ought to believe, but without affecting the epistemic status of the belief.

According to epistemic purism, *one should believe something if and only if has adequate evidence for what one believes* (Marušić, 2011, pp. 33–34). Such a position is grounded in the idea that what we ought to believe depends only on truth-relevant considerations that indicate that a belief is true (Gardiner, n.d.), and evidence is such a guide to truth (Conee & Feldman, 2004). Factors other than evidence do not increase our chances of getting to the truth, and if they do, it is purely coincidence or chance. For instance, if the state agency director wonders whether they should believe that the medicine cures cancer, they will ground their belief in evidence provided by scientific research, not in the wishful thinking of the families of the sick.

On the other hand, there is reasons pragmatism, which assumes that we may ask the question of what we should believe not only from the epistemic but also from a pragmatic or moral point of view. Therefore, what we should (pragmatically) believe depends on non-truth-relevant considerations: pragmatic factors such as convenience or utility or moral factors. Therefore, sometimes we can or should (pragmatically) believe X without sufficient evidence, or even against the evidence (Chignell, 2018, sec. 6.1). For example, we can argue that in war, the defenders should believe in the weakness of the attackers in order to maintain high morale, even if the attackers have a much larger army. There are more radical versions of reasons pragmatisms, such as Rinard’s hard pragmatics according to which all reasons are pragmatic, also the alethic ones (e.g. Rinard 2019). But there are also so-called moderate pragmatists, such as Quanbeck (forthcoming), who believe that pragmatic reasons are just distinct from the alethic ones.

## Evidence-Based Regulation and Precautionary Principle

The perspective of ethics of belief allows me to interpret concepts of EBR and PP as normative standards of lawmaking institutions’ belief, representing epistemic purism and reasons pragmatism (dEBR and dPP accordingly).

Evidence-based regulation (EBR) refers to the approach of grounding particular practice-based disciplines in the best available, up-to-date scientific evidence, loosely inspired by the evidence-based medicine movement. Other representations of this developing trend are, e.g. evidence-based management or evidence-based education.

Therefore, EBR is a normative postulate according to which the regulatory process should rely on scientific evidence, research and data to inform and guide the development of laws and policies (see Rachlinski, 2011; van Gestel & de Poorter, 2016). This approach emphasizes the use of empirical data and systematic research to identify and address social and public policy problems and to evaluate the effectiveness and outcomes of different policy options. EBR aims to ensure that policies are informed by the best available evidence, rather than by political ideology, personal beliefs or special interests. As Meßerschmidt (2020, p. 18) describes it, ‘[l]egislation should not be based on assumptions and hopes but on determined facts and well-founded forecasts’. Many legal scholars call for an evidence-based turn in particular fields of law (e.g. Ulrich, 2016; White & Willmott, 2019; Nicolosi, 2022)

EBR has gained a lot of interest in recent years, not only in academic literature but also in legal practice. For instance, since 2002, the European Commission (2021) has been advancing the Better Regulation agenda. It is a comprehensive initiative aimed at improving the quality and effectiveness of EU legislation. The agenda focuses on making EU regulations more evidence-based and transparent while reducing the regulatory burden on businesses and citizens. Its goal is to ensure that EU legislation is informed by the best available evidence and is effective in achieving its intended objectives. To achieve this, the EU employs various tools such as impact assessments, public consultations and ex-post evaluations to inform the development and implementation of regulations. Evidence-based approach manifested in the Better Regulation agenda aims to increase public trust in the EU legislation (Guéguen & Marissen, 2022, p. 31).

I am not going to analyse in detail how the EBR is applied in particular legal systems or whether it is effective. As mentioned in the problem outline, I will be interested in the normative standard of dEBR, which I treat as an application of epistemic purism in thinking about legal regulation. Moreover, I assume that it is a correct approach when it comes to law-making. For example, the lawmaking institutions should not believe that anthropogenic climate change is a fact without sufficient evidence supporting it and on the contrary—from the perspective of evidentialism, they ought to believe in anthropogenic origins of climate change if there is a consensus of scientists about it. And before forming a belief in that matter, they should gather and analyse the evidence provided by science. What is important, it does not determine in any particular way how the lawmaking institutions should act upon this belief (which means that EBR and dEBR are distinct normative concepts). Knowledge-action link is another matter, dependent on many reasons—moral, economic or political. In the paper, I am interested solely in the norms of ethics of belief and the question of the grounds for lawmaking institutions’ beliefs.

The PP, on the other hand, expresses popular intuition ‘better safe than sorry’ and has its roots in German Vorsorgeprinzip — a founding principle of German environmental law from the 1970 s. However, similar arguments were used even earlier, in the 1950 s in the USA about fluoridation of water or in the 1960 s in Europe concerning nuclear power plants (Ahteensuu & Sandin, 2012, p. 965). Nowadays, the PP is one of the most important principles in environmental law, public health law, pharmaceutical law and food safety law, especially in the EU. Among many different formulations of the PP, one of the most influential is the Wingspread Statement of Precautionary Principle (1998) according to which ‘when an activity raises threats of harm to human health or the environment, precautionary measures should be taken even if some cause-and-effect relationships are not fully established scientifically’.

In the philosophical literature, as well as in public discourse, there are various interpretations of the PP. Some claim that it is a decision rule (e.g. Hansson (1997) with his maximin decision rule) guiding what action should be chosen or not chosen. Others interpret it as a procedural norm specifying how such choices should be made (e.g. United Nations Conference on Environment and Development, 1992). There are also positions trying to integrate action-guiding, epistemic and procedural aspects of the PP (e.g. Steel, 2015). As this paper focuses on beliefs rather than actions or procedures, I adopt Martin Peterson’s epistemic interpretation according to which PP is a belief-guiding principle ‘characterised in terms of what it urges us to believe’ (Peterson, 2007, p. 5). dPP formulated in such a way does not say what decision one should make, what action one should take or what kind of procedure one should apply. It just says what beliefs should one hold.

Although both dEBR and dPP feature prominently in contemporary legislation, they seem to conflict with each other. The dEBR (epistemically) allows a legislator to claim X only if there is scientific evidence for X, while the dPP (pragmatically) allows a legislator to claim X even if there is no scientific evidence for X. However, in the end, the legislator has to decide what they should believe (all-things-considered) in order to turn it into a regulation. It is similar to the situation where one is epistemically justified in believing that p (according to epistemic purism), but pragmatically justified in believing that not-p (according to reasons pragmatism), but needs to decide what to believe in order to implement that belief in real life. In the paper, I suggest that such a conflict may be only apparent and can be explained by appealing to the concept of encroachment. The pragmatic, moral, or political considerations will influence already what one should believe at the epistemic level.

## Pragmatic Encroachment

Let us start with the concept that emerged first. Pragmatic encroachment understood in terms of ethics of belief is a middle-way theory which incorporates evidentialist intuition that what we should believe is determined by justification based solely on the evidence (as in epistemic purism) with a pragmatic intuition that non-alethic considerations are sometimes impossible to avoid. However, according to the version of pragmatic encroachment, I assume in the paper, these non-alethic reasons only affect the amount of evidential support needed for a belief to qualify as epistemically justified, i.e. the *evidential threshold*. In other words, pragmatic reasons may encroach on the epistemic by influencing how much evidence we need to be justified in holding a belief. Bolinger (2020b, p. 13) calls this kind of encroachment ‘threshold raising’. It is important to note that in the case of pragmatic encroachment, pragmatic reasons have an indirect effect on the epistemic sphere, rather than a direct effect on the pragmatic sphere, as reason pragmatism proposes.

There is an independent but related discussion in epistemology between invariantists and contextualists that will be useful later. This discussion centres on whether the standards for knowledge are fixed or vary depending on the context in which the knowledge attribution is made. Invariantism and contextualism are two opposing views in epistemology. The former is the traditional view that the truth conditions of knowledge claims do not vary with context. In other words, whether someone knows something is determined by fixed standards that do not change depending on factors such as the speaker or audience. The latter states that the truth conditions of knowledge claims can vary with context. According to this view, whether someone knows something can depend on factors such as the standards of evidence in play and what is at stake for the attributor. As Ichikawa and Steup (2017, sec. 13) note, ‘contextualism and pragmatic encroachment represent different strategies for addressing some of the same'shifty'patterns of intuitive data‘. Although contextualism was the original theory, pragmatic encroachment theorists were inspired by it and attempted to explain similar patterns without making the same semantic commitments. While they may be considered rival theories, contextualism and pragmatic encroachment are not necessarily contradictory. It is possible to maintain that justification requires different standards in different contexts while also taking into account the subject’s pragmatic situation when determining if those standards are met.

The most notable illustration of pragmatic encroachment was authored by Keith DeRose (1992, p. 913). It is worth mentioning that it was presented as part of DeRose’s argument for moderate contextualism (and against radical contextualism):

Bank Case A (Low Stakes). My wife and I are driving home on a Friday afternoon. We plan to stop at the bank on the way home to deposit our paychecks. But as we drive past the bank, we notice that the lines inside are very long, as they often are on Friday afternoons. Although we generally like to deposit our paychecks as soon as possible, it is not especially important in this case that they be deposited right away, so I suggest that we drive straight home and deposit our paychecks on Saturday morning. My wife says, ‘Maybe the bank won’t be open tomorrow. Lots of banks are closed on Saturdays.’ I reply, ‘No, I know it’ll be open. I was just there two weeks ago on Saturday. It’s open until noon.’

Bank Case B (High Stakes). My wife and I drive past the bank on a Friday afternoon, as in Case A, and notice the long lines. I again suggest that we deposit our paychecks on Saturday morning, explaining that I was at the bank on Saturday morning only two weeks ago and discovered that it was open until noon. But in this case, we have just written a very large and important check. If our paychecks are not deposited into our checking account before Monday morning, the important check we wrote will bounce, leaving us in a very bad situation. And, of course, the bank is not open on Sunday. My wife reminds me of these facts. She then says, ‘Banks do change their hours. Do you know the bank will be open tomorrow?’ Remaining as confident as I was before that the bank will be open then, still, I reply, ‘Well, no. I’d better go in and make sure.’

In Bank Case A, it appears that DeRose requires less evidence to justify his belief about the opening hours of the bank. This is because any potential negative effects resulting from an erroneous belief are minimal. Conversely, in Bank Case B, DeRose must possess a greater amount of evidence to hold a belief regarding the bank’s opening hours due to the high stakes involved; if he were to be incorrect, the consequences would be severe. The stakes are understood here as potential negative effects caused by one’s false belief. As Jaakko Hirvelä (2023, p. 3) notices, ‘[t]he key motivation behind pragmatic encroachment is the idea that epistemic justification is intimately connected to action: you are justified to believe that p only if you can (rationally) act as if p were the case‘. As a result, proponents of pragmatic encroachment assert that the higher the stakes are, the greater the evidential threshold for justification becomes. Nevertheless, this remains a strictly evidentialist position. In both cases, DeRose still relies solely on the evidence to form his belief. The pragmatic factors, or stakes, do not influence the justification directly (they do not operate as evidentiary reasons) but merely affect the amount of evidence needed to justify his belief.

Renee Bolinger (2020b, p. 8) distinguishes between cautious and robust encroachment.^2^ Caution encroachment operates in the same way as described above, with the threshold of evidence being directly proportional to the high stakes (let me refer to this as a positive encroachment). Thus, if the stakes are high, the threshold is also high. In other words, if the consequences of having a false belief are serious, then we need more evidence to support the belief. According to robust encroachment, high stakes can be sometimes inversely proportional to the threshold. In such a case, if the stakes are high, the threshold is lowered (let me refer to this as negative encroachment). In other words, if the consequences of having a false belief are serious, then we need less evidence to support the belief. An example of robust negative encroachment is the initial trust in the testimony of victims of sexual violence (see Lloyd 2022 but contra Leary 2023; Gardiner 2021a; n.d.). In such cases, the stakes are generally high, including the victim’s psychological well-being and encouraging other victims to come forward. There are good reasons however to lower the evidential requirements and prima facie believe the victims, even if it is one person’s word against another and there is no other evidence available^3^.

## Moral Encroachment

More recently, philosophers working on ethics have pointed out that not only the pragmatic domain can encroach on our beliefs. Moral encroachment is a similar view to pragmatic one (Fritz, 2017; Moss, 2018, p. 193; Schroeder, 2018, p. 117; but cf. 12–13 Basu, 2019). As David Enoch (2016, p. 35) points out ‘A natural way of extending thoughts of pragmatic encroachment is to think about how what is morally at stake can affect the relevant epistemic standards’. While pragmatic encroachment refers to a wide range of non-epistemic considerations, moral encroachment focuses on complementing the evidentialist position with moral considerations. It tackles the problem of whether it is always morally permissible to believe something epistemically justified or maybe sometimes we should withhold or even reject epistemically justified belief just because it would be morally wrong to hold it (or contrary: maybe we should accept the belief which is not epistemically justified just because it would be morally right).

Moral encroachment is often illustrated in the literature with the Cosmos Club example, originally presented by Tamar Szabó Gendler (2011, p. 35):

In the summer of 1995, historian John Hope Franklin - author of From Slavery to Freedom — received a call from the White House informing him that President Clinton planned to present him with the Presidential Medal of Freedom, the nation’s highest civilian honor. On the night before the award ceremony, Franklin hosted a dinner for a small group of friends at the Cosmos Club, a Washington DC social organization of which he was a member. He writes: ‘It was during our stroll through the club that a...woman called me out, presented me with her coat check, and ordered me to bring her coat. I patiently told her that if she would present her coat to a uniformed attendant, “and all of the club attendants were in uniform”, perhaps she could get her coat’.

Gendler believes that it is an example of conflict between epistemic and moral norms, concluding that while the woman correctly followed the epistemic norm, she also violated a moral norm. The adherents of moral encroachment claim that believing that a Black man in a club is an attendant based on his race is morally wrong and that this moral wrongness makes the situation high-stakes. They argue that the moral reasons against forming the belief in such a way are so significant that the woman’s evidence is not enough to make her belief acceptable from an epistemic standpoint. She should have collected more evidence (positive moral encroachment). However, there is disagreement among advocates of moral encroachment about which specific moral features of the context are responsible for this effect. For instance, Basu (2019) and Schroeder (2018) refer to the risk of *doxastic wronging*, Bolinger (2020a) to the risk of magnifying the wrongs associated with mistakenly assuming stereotypes by repetition and Moss (2018) to the psychological harms of negative stereotyping.

Even though moral encroachment has been recently discussed mainly in the contexts of doxastic wronging or harmful generalization based on statistical evidence, I would like to refer to a more general sense of moral encroachment, according to which the moral factors determine when we speak about high or low stakes and this way, they influence the threshold of evidence. As noticed by Rima Basu (2021, p. 104), moral encroachment corresponds to the concept of *inductive risk*, widely discussed in the philosophy of science but also the literature about the PP. The notion of inductive risk can be traced back to Richard Rudner (1953) who pointed out that being a scientist requires accepting or rejecting hypotheses. When deciding how strong the evidence needs to be to make such a decision, the scientist must take into account the ethical significance of making a mistake. Rudner used the example in which we require an elevated level before accepting that a potentially lethal drug is safe, but in the case of a machine that stamps belt buckles, we would need a less stringent level of confirmation or confidence to determine whether it is defective. The difference between these cases is the severity of the moral consequences of making a mistake. Therefore, while assessing how much evidence we should have to be justified to believe X, we ought to take into account the moral costs of being wrong (or sometimes of being right^4^), e.g. whether any people will be harmed, the environment will be damaged or any groups will be wronged.

## Why Encroachment Works and Purism Does Not

The question remains, however, what makes the encroachment approach more compelling than epistemic purism, according to which what we should believe epistemically should remain independent of moral, pragmatic or political considerations. To answer this question, I will examine some of the positions critical about encroachment. This will clarify the core disagreements in the debate and lay the groundwork for understanding why the encroachment perspective offers a promising account of what we (epistemically) should believe.

An influential attempt to resist encroachment views, while maintaining sensitivity to practical and moral context, is developed in Georgi Gardiner’s (2018; n.d.; 2021b) work on epistemic risk and (epistemic) purism. She proposes a reformulation of epistemic justification through a relevant alternatives framework (Gardiner 2021b) that departs from the dominant probabilistic model, which treats justification as a matter of degree based on numerical evidential thresholds. On Gardiner’s account, whether a belief is (epistemically) justified depends on whether the agent can rule out relevant possibilities of error, with relevance determined contextually. As the practical or social stakes increase, so does the range of alternatives that must be epistemically addressed. In high-stakes situations, agents are expected to rule out more remote or socially significant error alternatives than in low-stakes contexts. Crucially, however, Gardiner maintains that this shift in relevance does not mean that epistemic justification is influenced by non-epistemic values; the standards remain evidentialist, governed by the agent’s ability to rule out error in light of the available evidence.

Gardiner’s framework is particularly responsive to the kinds of cases that have motivated both pragmatic and moral encroachment. She argues that many beliefs criticised by encroachers — such as those based on racial or gender generalisations — are already epistemically unjustified because they fail to rule out alternatives that are morally and socially salient (Gardiner n.d.). Drawing on insights from virtue and social epistemology, Gardiner further argues that the moral status of a belief often depends on the understanding in which it is embedded (Gardiner 2018). Beliefs formed through epistemically negligent or morally indifferent reasoning may lack justification not only because of their propositional content, but also because of the broader epistemic stance they reflect — one that may ignore individualising evidence, rely on stereotypes or dismiss the testimony of marginalised groups. Her account thus offers a nuanced picture of how epistemic agents navigate risk without collapsing epistemic norms into moral ones.

From a non-purist perspective, Gardiner’s risk-sensitive relevance framework is indeed a welcome improvement over rigid evidentialism. However, her attempts to explain away the need for encroachment by appealing to a more expansive evidentialist framework risk bringing in moral and practical considerations by the back door. In Gardiner’s case, the appeal to context-sensitive relevance — while nominally limited to epistemic factors — ends up tracking many of the same features that encroachment advocates claim directly affect epistemic justification: social salience, power asymmetries and the costs of being wrong in morally charged contexts. But what makes them relevant is often a moral concern, such as respect, trust or relational vulnerability. I believe it would be more transparent and normatively honest to recognise that moral considerations may influence epistemic justification, rather than to reclassify them as epistemic by widening the evidence net.

Moreover, while Gardiner rightly rejects a fixed threshold for justification, she maintains that the threshold should be determined only by truth-relevant epistemic factors. However, in many real-world cases — such as forming beliefs based on statistical generalisations about marginalised groups — the very decision about which alternatives to treat as relevant is morally charged. While Gardiner advocates careful attention to context, individualising evidence and testimonial nuance, such practices are often undermined by structural inequity, implicit bias and testimonial exclusion. Under these conditions, what counts as ‘available’ or ‘relevant’ evidence is not epistemically neutral — it is often shaped by unjust social structures. In such cases, moral stakes cannot merely affect the context of inquiry — they must affect the epistemic justification of belief itself.

Robert Carry Osborne (2021), on the other hand, tries to shift the debate from the realm of epistemic justification to that of interpersonal ethics. Rather than attempting to reconcile moral and epistemic norms within a unified framework, Osborne distinguishes between them by arguing that beliefs may be epistemically justified in themselves, but may still be morally objectionable because of how they reflect one’s attitudes or evaluative stance towards others. According to this view, what we owe to each other is not necessarily to refrain from forming certain beliefs, but rather to maintain an appropriate moral regard for others. This regard is primarily non-doxastic: it consists in how we treat and relate to others, rather than in what we believe about them per se. On this account, the problem with morally troubling beliefs — such as suspecting a loved one of wrongdoing or relying on racial generalisations — is not that they are epistemically unjustified, but that they may express disrespect, distrust or a failure of moral regard. Strategy 2 thus aims to redirect the moral critique of belief away from epistemic norms and towards moral norms governing interpersonal attitudes.

However, Osborne’s strategy offers only a narrow and ultimately inadequate response to the challenges posed by encroachment views. First, it does not respond at all to pragmatic encroachment, where the stakes of belief are not moral in the sense of interpersonal respect, but practical — such as financial risk, physical harm or decision-making under uncertainty. Second, Osborne’s position is structurally limited to the domain of interpersonal morality, focusing on how we see and treat others — especially in close relationships — while leaving unaddressed the broader moral dimensions of belief in public, institutional or structural contexts. Many morally objectionable beliefs do not simply reflect personal disregard but contribute to systemic harms that are not captured by Osborne’s emphasis on interpersonal regard. Second, this strategy of encroachment argues that beliefs formed in high-stakes contexts, whether moral or practical, may justifiably require more evidence or higher epistemic standards because of the costs of being wrong. Finally, even within the moral domain, moral encroachment theorists contend that Osborne’s account captures only a subset of the normative pressures at play — namely, cases of dogmatic wrongdoing, where the belief expresses a morally inappropriate attitude. But moral encroachment is about more than expressive ethics; it is about how the stakes of being wrong in morally salient contexts can raise the evidential requirements for justified belief. Osborne’s strategy thus preserves epistemic purism only by narrowing the scope of moral evaluation and overlooking how deeply morality and risk can normatively penetrate epistemic life.

A related defence of purism is developed by Jaakko Hirvelä (2023), who focuses on the structural consequences of moral encroachment rather than its intuitive force. He also distinguishes between moderate and radical versions of the view, the latter holding that moral facts can directly determine whether a belief is epistemically justified. Hirvelä argues that this position leads to several troubling implications: the failure of epistemic closure, the dynamic nature of justification (where a belief can become unjustified without any change in the evidence) and the possibility that some true propositions may be epistemically unjustifiable. Most importantly, he warns that radical moral encroachment would allow moral norms to override epistemic norms, giving morality lexical priority in doxastic justification. As an alternative, Hirvelä defends a purist model that preserves the independence of epistemic and moral norms. A core element of his view is the rejection of positive epistemic obligations: he denies that agents are ever obliged to believe what their evidence supports. On this account, belief may be epistemically permissible, but agents may still be morally or practically required to suspend judgment in high-stakes cases — thus resolving the tension without altering the criteria for justification.

The most problematic feature of Hirvelä’s account is precisely this rejection of epistemic duties. The claim that agents are never required to believe what their evidence supports is not only controversial, it also threatens to undermine the normative force of epistemic norms. If epistemology only permits belief but never requires it, then the distinction between epistemic responsibility and epistemic indifference becomes blurred. Moreover, by shifting normative pressure entirely to moral or practical considerations, Hirvelä’s view risks rendering epistemic norms normatively inert in precisely those situations where belief is most consequential. In morally or pragmatically high-stakes contexts, the question is not simply whether belief is allowed, but whether it is epistemically inappropriate if it does not meet heightened standards of justification. Denying the existence of epistemic duties thus avoids, rather than answers, the need for a framework in which stakes can directly influence justification, and thus the role of epistemology in guiding responsible belief.

What makes encroachment a particularly promising strategy is that it offers a way to reconcile the normative appeal of both evidentialist and pragmatic intuitions without fragmenting the structure of epistemic justification. Most of the purist responses in the literature focus narrowly on moral encroachment, often in its radical form associated with doxastic wrongs — cases in which beliefs are criticised for what they say about the believer’s moral regard for others. But this captures only a small subset of the relevant phenomena. Moral encroachment, properly understood, involves a much wider range of reasons — such as the risk of harm, structural injustice or institutional responsibility — that raise the stakes of belief and hence the evidential threshold. And moral encroachment is only part of the picture: pragmatic and, as the next section will show, political encroachment raises parallel concerns. If we adopt the purist framework, then the tension between epistemic and pragmatic reasons re-emerges at the level of practical reasoning, leading to potential conflicts in what we ought to believe all things considered. But the very existence of an all-things-considered epistemic ought is itself contested (Kauppinen 2023; Quanbeck & Worsnip forthcoming), and such conflicts undermine the action-guiding role of epistemic norms. The encroachment approach, by contrast, offers a more unified framework: it preserves evidentialism as the core of epistemic justification while allowing the evidential threshold to be sensitive to practical, moral or political stakes. In doing so, it explains apparent conflicts not by weighing epistemic and non-epistemic reasons against each other, but by recognising that the stakes themselves can shape what counts as justified belief.

## Political Encroachment

As we see, morality can encroach on the epistemic. But if political normativity can be distinguished from moral normativity, then we should be able to talk not only about moral but also about political encroachment.^5^ The distinction between moral and political normativity comes, e.g. from Bernard Williams (2009; see also Bermejo-Luque 2024), who argues that political normativity emerges from practical deliberation within a political community, rather than being derived from moral principles. While moral considerations influence political decisions, they do not exclusively determine them, allowing political normativity to operate independently in its domain. This separation allows for a distinct political normativity that is not subordinate to moral imperatives, thus emphasising the autonomy of political philosophy from moral philosophy. In his critique of political moralism, Williams is committed to political realism. For him, political value, such as legitimacy, is determined by how well political action addresses the *first political question* of securing order, protection and cooperation.

More recently, Javier Rodríguez-Alcázar (2017) has proposed a third way between political moralism and political realism. He criticises both political moralism and political realism for their common assumption that politics must have substantial, permanent goals or constraints, such as justice, freedom or order. Instead, the author proposes *political minimalism*, which defines politics as a form of collective instrumental rationality that aims to provide adequate means to achieve the diverse goals pursued by a political community.

Political minimalism avoids imposing a single, fixed goal on politics and instead focuses on answering the question ‘What shall we do?’ in a way that is acceptable to all members of the community. This approach emphasises the *reciprocal containment* of morality and politics: from a political perspective, politics embraces morality by considering the plurality of moral codes as social facts, while from a moral perspective, individuals can evaluate political actions based on their own ethical beliefs. By shifting the focus of political philosophy from prescribing ends to facilitating the realisation of people’s ends through effective means, political minimalism offers a flexible, context-sensitive approach that avoids the pitfalls of both moralism and realism while maintaining a normative framework for evaluating political action.

Consider a border control agency deciding whether to believe that a person who has illegally crossed the border has been oppressed in his or her home country, which would make him or her eligible for asylum. From an epistemic perspective, the government should only believe this claim if there is sufficient evidence, such as credible documentation or corroborating testimony, to ensure that the belief is well-founded and accurate. From a moral perspective, the high stakes of being wrong — such as denying asylum to a genuine refugee and exposing them to further harm — might lower the evidential threshold (negative moral encroachment). Compassion and a duty to protect vulnerable individuals might justify believing the claim even with limited evidence, as the moral cost of a false negative (rejecting a genuine claim) is unacceptably severe. Conversely, from a political perspective, the high stakes of potential consequences of being wrong could raise the threshold of evidence. Granting asylum (on a massive scale) to people who are in reality only economic migrants or granting asylum to an undercover agent of foreign services could cause public backlash against illegal immigration, risk of growing anti-immigrant populism or excessive state spending. Political considerations, such as maintaining border security or managing public opinion, may require more robust evidence to justify the belief, even if the epistemic evidence is sufficient because the political costs of a false positive (accepting a false claim) are deemed too high. This illustrates how the justification for belief can vary across epistemic, moral and political frameworks, reflecting the complex interplay between evidence, ethics and political realities.

## Doxastic Precautionary Principle as Political Encroachment

Bermejo-Luque & Javier Rodríguez-Alcázar (2023) have recently applied political minimalism to the discussion on precaution and formulated the PP as a political principle. According to their interpretation, the PP should not be understood as a moral imperative, but as a political norm that balances the moral obligation to avoid unwanted risks with the diverse goals and values of a political community. My approach follows this line, but at the doxastic level, where the epistemic encounters the pragmatic, the moral and the political.

Assuming the dEBR, the beliefs underlying regulations should be grounded solely in epistemic considerations. However, taking into consideration conflicting values within the society, it seems that lawmaking institutions should also consider other consequences of their beliefs. Stephen John (2019, p. 4) illustrates it with the example of the BSE crisis in the 1990 s when the UK lawmaking institutions had to make decisions about whether or not to believe the connection between Bovine Spongiform Encephalopathy in cows and Creutzfeldt-Jakob disease in humans. Such beliefs were guiding such political dilemmas as whether to slaughter millions of cattle or whether to ban beef export due to the risks to public health. Recently, governments all over the world were concerned about whether to believe in various facts about the coronavirus, such as its lethality or whether vaccines stop its transmission. To adopt beliefs, one has to consider the risks of both accepting false claims and rejecting true claims. John (2010, p. 4) rightly points out that lawmaking institutions also require specific standards to assess such claims. He calls these standards *acceptance principles*, which, I assume, are exactly the kind of norms of ethics of belief for lawmaking institutions.

John believes that lawmaking institutions should adopt *floating acceptance principles*. He actually has in mind norms guiding the threshold-raising encroachment (he is even evoking DeRose’s bank cases) without calling it like that. John (2019, p. 5) argues that

[t]he degree-of certainty that some claim should enjoy before it is properly accepted by policy-makers should vary with the expected costs of acting on different sorts of error. The higher the expected costs of acting on ‘false positive,’ the more evidence policy-makers should require before accepting the relevant claim; while the higher the expected costs of not acting (on the basis of a ‘false negative’) the less evidence policy-makers should require for acceptance. This principle instantiates a basic norm of practical reasoning: that the amount of evidence it is rational to demand before accepting a claim turns on the relative costs of false acceptance and of failures to accept true claims

I believe that such a floating acceptance principle is suggested exactly by political encroachment, which claims that political factors often determine whether the stakes are high. In the case of the ethics of belief for lawmaking institutions, this determination will result from practical discourse involving weighing relevant values. This approach aligns with the idea that the PP operates within the realm of collective instrumental rationality, as proposed by Rodríguez-Alcázar, providing a robust framework for managing uncertain risks in a politically legitimate manner.

Returning to the previously discussed example, if the regulatory body is considering whether to apply the dPP, which could entail the slaughter of millions of cows... they need to take into account the possible costs of being wrong as a result of decisions based on such beliefs. Lawmaking institutions should consider the different (usually conflicting) values and interests that may be infringed upon, such as the well-being of cows, public health, respect for local tradition and economic liberty, among others, and *exercise of collective instrumental rationality* to answer the question ‘What shall we believe?’ to paraphrase Rodriguez-Alcazar’s (2017) question ‘What shall we do?’

Furthermore, political reasons not only determine the stakes but also the direction of encroachment, i.e. whether the high stakes raise or lower the threshold of evidence. It is disputable if there is a default direction of the dPP, but it can reasonably be interpreted both ways, as positive (‘The higher the stakes, the more evidence about X’s safety we need’) or negative (‘The higher the stakes are, the less evidence about X’s dangerousness we need’) encroachment on the dEBR, depending on different contexts (see discussion between Carter and Peterson (2015), Steglich-Petersen (2015) and Carter and Peterson (2016)). Additionally, a different direction of the dPP often involves a difference in placing the burden of proof: on the proponent of the potentially dangerous activity or the lawmaking institutions. As Steve Wexler (1999, para. 2) puts it, ‘burden of proof and benefit of the doubt are opposite sides of the same coin’.

As explained in the Communication from the European Commission (2000, p. 20), the dPP is often applied through prior approval procedures before certain products are placed on the market, such as drugs, pesticides or food additives. This shifts the responsibility for producing scientific evidence onto the business community. In such cases, the burden of proof is reversed, requiring that substances be considered hazardous until proven otherwise. This occurs particularly when substances are deemed a priori hazardous or potentially harmful to human life, health or the environment (which are the important values that EU environment or food law aims to protect). A good illustration can be found in the EU regulation on GMOs, in which the PP is positive political encroachment, shifting the burden of proof due to the concern that GMOs may pose unknown risks to human health and the environment, and that it is better to err on the side of caution (see Myhr, 2010). However, when a prior approval procedure does not exist, it may be up to private individuals, consumer associations or public authorities to demonstrate the level of risk posed by a product or process. This fits the formulation of the dPP, as negative political encroachment, which promotes such values as innovativeness and economic liberty.

## Summary

At the outset of the article, I posed the question of whether it is possible to reconcile two opposing conflicting demands understood in epistemic terms: evidence-based regulation and the precautionary principle. These demands are manifestations of ethics of belief for lawmaking institutions: the former being epistemic purism, while the latter is reasons pragmatism. I have shown that in the literature, the dPP has already been interpreted as a pragmatic encroachment in its threshold-raising version. This implies that pragmatic reasons may raise (in case of positive encroachment) or lower (in case of negative encroachment) the threshold of evidence required to justify the belief that X presents a danger that requires precautionary measures (such as prohibiting X). Such a solution resolves the tension between evidentialist and pragmatic intuitions at the very epistemic level, without the need to consider all-things-considered ought to believe.

My thesis is that the dPP should actually be interpreted as political encroachment on the dEBR. This means that while we accept the normative postulate that lawmaking should be based solely on evidence according to the dEBR, the dPP comes into play in cases where high stakes, such as human life or natural environment, are threatened, and there is scientific uncertainty about the nature and probability of these threats. The dPP then influences the evidence thresholds. But political reasons also determine the direction of political encroachment on dEBR, and consequently, the allocation of the burden of proof.

## Data Availability

Not applicable.

## Footnotes

1 For the purpose of this discussion, when I use the term ‘lawmaking institution’, I am referring to any entity, institution, group of people or individuals with the authority to create laws. This includes not only those at the national level but also at regional or transnational levels, such as the European Union. Regardless of the specific level of governance, lawmaking institutions are responsible for enacting and enforcing laws that govern the behaviour and interactions of individuals within a given jurisdiction. I do not address any metaphysical, socio-ontological or epistemological discussions regarding how lawmaking institutions possess the ability to hold beliefs, examine the evidence or hold values. In particular, I do not commit myself to any realistic, eliminativist or fictionalist theories of collective beliefs (see Poslajko, 2023). Instead, I assume that the lawmaking institutions can be treated as if they are individuals in terms of asserting, abandoning and holding propositional attitudes of some kind (for which, without entering into about their nature, I use the general term *beliefs*). Therefore, please treat all the examples which concern the individuals as referring analogously to lawmaking institutions.

2 Even though she refers to cautious and robust encroachment, I believe this distinction can be applied to pragmatic encroachment as well.

3 It is important to note however that the robust encroachment is a controversial position; Pace (2011) or Mitova (2024) accept it and Schroeder (2018, p. 126) denies it.

4 Interesting problem, which deserves careful consideration, was noticed by Basu (2021, p. 105), who claimed, even though ‘theories of inductive risk have focused naturally on the risk of drawing false conclusions, of getting things wrong […], we ought also to consider is that which comes from getting things right.’ Let us imagine a situation in which scientists provide us with the evidence that homosexuals are responsible for transmitting a deadly virus to heterosexuals (and heterosexuals do not). Moral reasons, such as danger of discrimination or stigmatization, make the stakes exceedingly high. In such a case, the lawmaking institutions should consider whether such evidence is enough accepting a belief that the homosexuals pose a threat to heterosexuals and there is a need to take preventive steps. The adherent of moral encroachment may argue that taking into account the possible harms of scientists being right, we should not accept such belief. This is however problematic from a point of view of threshold-raising account of encroachment. What amount of evidence and what level of confidence would be enough for the lawmaking institutions to finally accept the claim? Or is it infinitely high? I think the issue can be more threshold-raising account of encroachment addressed by the adherents of sphere-expanding moral encroachment (Bolinger, 2020b, p. 13). However, I will not elaborate on this here (but see Elliott & Richards, 2017, p. 265).

5 I would like to thank one of the anonymous reviewers of Acta Analytica for his important and inspiring comments and suggestions on political interference. I must admit that they were so convincing that I included them in the text, which I believe has benefited greatly from them.
